# *Toxoplasma gondii* seropositivity and the associated risk factors in sheep and pregnant women in El-Minya Governorate, Egypt

**DOI:** 10.14202/vetworld.2020.54-60

**Published:** 2020-01-09

**Authors:** Abdelbaset E. Abdelbaset, Maha I. Hamed, Mostafa F. N. Abushahba, Mohamed S. Rawy, Amal S. M. Sayed, Jeffrey J. Adamovicz

**Affiliations:** 1 Department of Animal Medicine (Clinical Laboratory Diagnosis), Faculty of Veterinary Medicine, Assiut University, 71526-Assiut, Egypt; 2 Department of Animal Medicine (Infectious Diseases), Faculty of Veterinary Medicine, Assiut University, 71526 Assiut, Egypt; 3 Department of Zoonoses, Faculty of Veterinary Medicine, Assiut University, 71526 Assiut, Egypt; 4 Department of Veterinary Pathobiology, College of Veterinary Medicine, University of Missouri, Columbia, Missouri 65211-5130, USA; 5 Department of Theriogenology, Faculty of Veterinary Medicine, Minia University, El-Minya, Egypt

**Keywords:** abortion, Egypt, risk factors, sheep, toxoplasmosis, zoonoses

## Abstract

**Background and Aim:**

The cosmopolite protozoan, *Toxoplasma gondii*, has a significant economic and medical impact. Cats traditionally play a predominant role in the disease maintenance cycle; however, humans can be infected as a result of milk and meat consumption of *Toxoplasma*-infected livestock. In addition, infected pregnant women, even symptomless, can pass the disease to their unborn fetus. The limited clinical records and absence of specific national educational programs in countries like Egypt underscore the need for periodic toxoplasmosis disease evaluation. Here, we identified *T. gondii* seroprevalence among sheep and pregnant women and the associated risk factors in El-Minya Governorate, Egypt.

**Materials and Methods:**

Using peripheral blood, we detected *T. gondii*-specific antibodies in 151 sheep and 96 pregnant women sera from El-Minya Governorate using latex agglutination and indirect enzyme-linked immunosorbent assay. The impact of different environmental and behavioral risk factors identified with in-person interviews and serology results on acquiring toxoplasmosis was statistically analyzed.

**Results:**

The overall toxoplasmosis seroprevalence was 39.1% and 22.9% in sheep and pregnant women, respectively. Significantly higher seroprevalence was correlated with increasing sheep age and geographical location. Nonetheless, no statistical significance was found based on abortion history and pregnancy status of the examined sheep. Exposure factors important for pregnant women included pregnancy trimester, contact with cats, and the habit of eating undercooked sheep meat, which all had a statistically significant association with *Toxoplasma* seropositivity.

**Conclusion:**

The current study confirms increased antibodies against toxoplasmosis in both sheep and pregnant women in El-Minya Governorate and a clear association between women’s age, contact with cats, and the habit of eating undercooked sheep meat and seroreactivity to *T. gondii*. These results strongly suggest the need for a more comprehensive epidemiological study and public health awareness education for toxoplasmosis.

## Introduction

Toxoplasmosis is a parasitic zoonosis caused by a cosmopolite protozoon, *Toxoplasma gondii*, one of the most ubiquitous parasites among humans and warm-blooded animals. It was reported that 30-50% of the human world population have been found infected with the disease, making *T. gondii* the most prevalent worldwide zoonosis [1]. The infection can be acquired through the ingestion of contaminated food or water with sporulated oocysts, consumption of undercooked meat harboring tissue cysts, and transplacental transmission of tachyzoites from mother to fetus [2]. In general, toxoplasmosis in humans is asymptomatic; however, postnatal infection results in fatal encephalitis in immunodeficient patients such as those with AIDS or organ transplant recipients [3]. Congenital toxoplasmosis, in addition, may cause spontaneous abortion, premature birth, and stillbirth in pregnant women who become infected for the 1^st^ time [4,5]. In sheep, *T. gondii* infection is responsible for economic and reproductive problems, resulting in abortion and stillbirth [6]. In Egypt, the seroprevalence of *T. gondii* infection in pregnant women has been reported to range between 11.8% and 67.6% [7-12] and between 4.1% and 100% in sheep [8,11,13-15].

In spite of the substantial medical and economic implications of *T. gondii* in humans and animals, the epidemiological status of this parasite in several localities in Egypt, such as El-Minya Governorate, requires additional study. While the significance of this disease is important worldwide as calculated by disability-adjusted life years, the situation in Egypt is unclear. In particular, the number of both human and animal abortion events associated with toxoplasmosis is not readily available. The overall economic impact effect on domestic livestock associated with toxoplasmosis is also unknown. Both of these variables have been determined in other countries, leading to better public health policies and practices. Therefore, the collection of critical data to better understand the impact of toxoplasmosis in Egypt is a significant unmet need.

The lack of current epidemiological data led us to perform this study to identify the seroprevalence of *T. gondii* infection among pregnant women and sheep and to assess the associated risk factors in El-Minya Governorate.

## Materials and Methods

### Ethical approval and informed consent

All procedures performed in studies involving human participants were in accordance with the ethical standards of Assiut University Institutional Ethical Committee. All pregnant women were informed about the study objectives, methodology, voluntary participation, and personal information confidentiality. Pregnant women were not literate enough to participate in a written consent and none of them agreed to sign a written consent due to the Egyptian culture. Therefore, verbal consents were taken from all adults and parental consents were attained before the participation of those under the age of 18. All animals were handled in compliance with the Assiut University regulatory rules for animal research. Furthermore, sheep samples and data were collected after the agreement of the owners. The study was approved by Assiut University Institutional Ethical Committee.

### Study area

**Figure-1:**
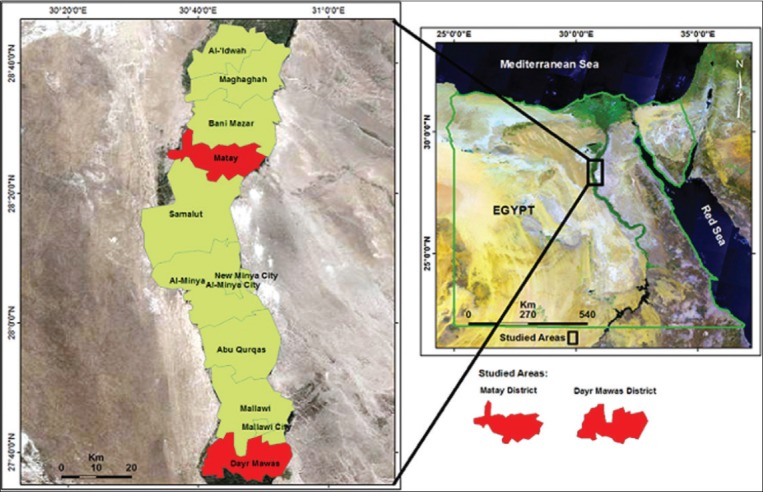
Satellite image of Egypt showing the general study locations. The survey covered different villages in Dayr Mawas and Matay districts, El-Minya Governorate, Egypt. [Source: The map was downloaded from http://www.gadm.org. ArcGIS 10.5 Desktop Software (Environmental Systems Research Institute, Inc., Redlands, California, USA) and GIMP 2.8.10 program (https://www.gimp.org/) were utilized in map processing].

The present study was conducted between 2017 and 2018 in El-Minya Governorate, one of the agricultural governorates which is located ~245 km south of the capital Cairo (Figure-1). At-risk population is 5.8 million and the residents rear sheep in small herds with limited range. The study focused on two districts (Dayr Mawas and Matay) which belong to El-Minya Governorate (Figure-1).

### Sample size determination

Because this is the first cross-sectional study to determine the seroprevalence of toxoplasmosis in sheep in El-Minya, to determine the sample size, we have used survey data reported previously in one of the nearest Egyptian governorates, El-Fayoum, where the seroprevalence in sheep using immunoglobulin G enzyme-linked immunosorbent assay (IgG ELISA) was found to be 98.4% [8]. We have selected that area for comparison due to similarities in the climatic parameters [16]. In the current survey, a sample size range of 24-42 sheep was a minimum based on the equation (n=Z^2^×P×q/d^2^), where “n” is the minimal calculated sample size and “Z” is a constant equal to 1.96 at 5% type 1 error or 2.58 at 1% type 1 error [17].

### Animal sera

A total of 151 serum samples were obtained from female sheep (mean age 3.9±1.9 years), from different villages belong to Dayr Mawas and Matay districts. Blood samples were collected by jugular vein puncture using sterile needles and Vacutainer tubes. Sample identity and relevant data, such as age, locality, pregnancy status, and abortion history, were recorded. Blood samples were immediately transported on ice to the laboratory at Animal Medicine Department, Faculty of Veterinary Medicine, Assiut University, and then centrifuged at 1800×g for 15 min to obtain sera [18]. Sera were kept in Eppendorf tubes and stored at −20°C until analysis.

### Human sera

A total of 96 pregnant women (mean age 26.3±6.6 years) were included in this study. Using a sterile syringe and needle, 5 mL of blood was taken by an experienced laboratory technician from each study participant. Blood samples were properly handled as described earlier. Participant data including name, age, locality, pregnancy, parity, exposure to soil, contact with cats, and consumption of undercooked meat for each participant were collected.

### Serological assays

Serum samples were screened using the latex agglutination test (LAT). LAT-positive samples were confirmed by IgG ELISA.

#### LAT

LAT was performed according to the kit manufacturer’s instructions (Toxo Latex Kit, CamTech Medical, UK). Briefly, 40 µL serum samples were placed on the kit agglutination slide. One drop of each negative and positive control sera was included for each assay. Twenty microliters of Toxo latex reagent were added to 40 µL sera samples into separate circles and mixed well with stirring sticks; then, the mixture was slowly rotated for 6 min. Samples were considered positive when agglutination was observed, which indicates that the concentration of antibody is equal to or more than 4 IU/mL.

#### IgG ELISA

Positive results observed by LAT were further confirmed using a commercial *Toxoplasma* IgG ELISA kit (Atlas Medical Company, UK). ELISA was performed following the manufacturer’s instructions. Anti-human enzyme conjugate was used for human sera, and anti-multispecies horseradish peroxidase conjugate was used (IDvet Innovative Diagnostics, Grabels, France) for sheep samples. The optical density was measured at 450 nm using Stat Fax 2100 Microplate Reader (Awareness Technology, Inc., FL, USA).

### Statistical analysis

To measure the impact of each factor individually on the occurrence of the disease in sheep (i.e., age, locality, pregnancy, and abortion history) and pregnant women (i.e., age, locality, trimester of pregnancy, parity, exposure to soil, contact with cats, and consumption of undercooked meat), Fisher’s exact test in GraphPad Prism 6.0 software (GraphPad Software, Inc., La Jolla, CA, USA) was used. Odds ratio (OR) and 95% confidence intervals were calculated. p<0.05 was considered statistically significant.

## Results

Initial screening of sera by LAT detected a toxoplasmosis seropositive rate of 42.4% (64 of 151) and 30.2% (29 of 96) in sheep and humans, respectively. ELISA test results further confirmed that 92.2% (59 of 64) of the LAT-positive reactor sheep and 75.9% (22 of 29) of the LAT-positive reactor pregnant women had *T. gondii* IgG antibodies (Table-1).

**Table-1:** Toxoplasmosis seroprevalence rate in sheep and humans.

Species	Latex agglutination test (screening)			Enzyme-linked immunosorbent assay (confirmation)		
	
	Number of tested	Positive number (%)	Negative number (%)	Number of tested	Positive number (%)	Negative number (%)
Sheep	151	64 (42.4)	87 (57.6)	64	59 (92.2)	5 (7.8)
Human	96	29 (30.2)	67 (69.8)	29	22 (75.9)	7 (24.1)

The overall seroprevalence of *T. gondii* among sheep was 39.1%. Among the analyzed variables in this study, age of the examined sheep and the sampling locality were statistically significant (p<0.0001, for both variables). A higher prevalence of *T. gondii* was detected in the 1-2-year-old group (66.7%, OR=75, 95% confident interval [CI]=4.0-1431) followed by 40.5% (OR=27, 95% CI=1.6-454) in >2-year-old age group; conversely, we did not detect any positive cases in <1-year age group. The prevalence of *T. gondii* was 57.4% in Matay (OR=14, 95% CI=5.1-38) in comparison to 8.8% in Dayr Mawas (OR=0.07, 95% CI=0.03-0.2). There was no significant effect (p>0.05) observed for abortion history and pregnancy status of the examined sheep (Table-2).

**Table-2:** Seroprevalence of *Toxoplasma gondii* antibody and the associated risk factors in sheep.

Factor	Number tested	Seroprevalence		Odds ratio (95% confidence interval)	p-value

		Positive number (%)	Negative number (%)		
Age (years)					
<1	19	0 (0)	19 (100)	Reference	<0.0001
1-2	21	14 (66.7)	7 (33.3)	75 (4.0-1431)	
>2	111	45 (40.5)	66 (59.5)	27 (1.6-454)
Locality					
Matay	94	54 (57.4)	40 (42.5)	14 (5.1-38)	<0.0001
Dayr Mawas	57	5 (8.8)	52 (91.2)	0.07 (0.03-0.2)
Pregnancy					
Pregnant	84	38 (45.2)	46 (54.8)	1.81 (0.9-3.5)	0.09
Non-pregnant	67	21 (31.3)	46 (68.7)	0.55 (0.3-1.1)
Abortion history					
Yes	3	2 (66.7)	1 (33.3)	3.2 (0.3-36)	0.6
No	148	57 (38.5)	91 (61.5)	0.3 (0.03-3.5)	

In pregnant women, the overall prevalence of *T. gondii* was 22.9%. Regarding the major risk factors associated with *T. gondii* seroreactivity, the pregnancy trimester showed statistically significant association with *Toxoplasma* seropositivity (p=0.025). Pregnant women at the 2^nd^ trimester were found to have the highest odds of exposure (OR=19, 95% CI=1-345) followed by those in the 3^rd^ trimester (OR=10, 95% CI=0.6-182). In addition, toxoplasmosis seropositivity was found significant (p=0.006) in relation to contact of the sampled women with cats and the odds of exposure were found higher in those who had contact with cats (OR=4.4, 95% CI=1.6-12) compared to those without previous contact with cats (OR=0.2, 95% CI=0.08-0.6). Moreover, the study showed that the habit of eating undercooked sheep had a significant effect on *Toxoplasma* seropositivity (p<0.0001). Higher odds of exposure were found among pregnant women with a habit of consumption of undercooked mutton (OR=14, 95% CI=4.2-44) compared to those without (OR=0.07, 95% CI=0.02-0.2). However, there was no significant association between age (p=0.7), locality (p=0.3), parity (p=0.1), or exposure to soil (p=0.2) and *T. gondii* seropositivity (Table-3).

**Table-3:** Factors associated with *Toxoplasma gondii* seropositivity among the pregnant women.

Factor	Number of tested	Seroprevalence		Odds ratio (95% confidence interval)	p-value

		Positive number (%)	Negative number (%)		
Age (years)					
17-19	12	1 (8.3)	11 (91.7)	Reference	0.7
20-24	32	9 (28.1)	23 (71.9)	4.3 (0.5-38)	
25-29	29	6 (20.7)	23 (79.3)	2.9 (0.3-27)	
30-35	15	4 (26.7)	11 (73.3)	4 (0.4-42)	
>35	8	2 (25)	6 (75)	3.7 (0.3-49)	
Locality					
Matay	46	13 (28.3)	33 (71.7)	1.8 (0.7-4.7)	0.3
Dayr Mawas	50	9 (18)	41 (82)	0.6 (0.2-1.5)	
Pregnancy					
1^st^trimester	16	0 (0)	16 (100)	Reference	0.02
2^nd^ trimester	28	10 (35.7)	18 (64.3)	19 (1.0-345)	
3^rd^ trimester	52	12 (23.1)	40 (76.9)	10 (0.6-182)	
Parity					
One time	28	3 (10.7)	25 (89.3)	0.3 (0.08-1.1)	0.1
Two times or more	68	19 (27.9)	49 (72.1)	3.2 (0.9-12)	
Exposure to soil					
Yes	33	5 (15.1)	28 (84.8)	0.48 (0.2-1.5)	0.2
No	63	17 (27)	46 (73)	2.1 (0.7-6.2)	
Contact with cat					
Yes	28	12 (42.9)	16 (57.1)	4.4 (1.6-12)	0.006
No	68	10 (14.7)	58 (85.3)	0.2 (0.08-0.6)	
Consumption of undercooked mutton					
Yes	18	12 (66.7)	6 (33.3)	14 (4.2-44)	<0.0001
No	78	10 (12.8)	68 (87.2)	0.07 (0.02-0.2)	

## Discussion

Sheep being important reservoirs for many pathogens with public health significance, our research group explored the epidemiological role of this small ruminant in toxoplasmosis. We have recently shown the presence of bacterial-specific antibodies against *Coxiella burnetii* and brucellae in sheep and humans residing in El-Minya Governorate with subsequent analysis of the associated risk factors [18,19]. Limited epidemiological data regarding toxoplasmosis in El-Minya Governorate warranted more research. In the current study, we examined toxoplasmosis as a serious foodborne infection that might affect pregnant women.

Our survey demonstrated a *T. gondii* seroprevalence of 39.1% in the examined sheep in El-Minya Governorate. This is the first report regarding toxoplasmosis in sheep in El-Minya Governorate, Egypt, and the findings are comparable with other regions in Egypt. By comparison, the current seroprevalence of *T. gondii* reported among sheep was similar to that in a previous survey conducted in six Egyptian governorates (Kafr El-sheikh, Matrouh, Giza, Menoufia, Sohag, and Qena), in which the seroprevalence was 37.7% [14]. Our seroreactivity results were higher than those reported in a recent study (2018) conducted in four governorates (Cairo, Giza, Dakahlia, and Sharkia), in which the seroprevalence ranged between 4.1% and 26% [15]. Conversely, *T. gondii* seroprevalence in sheep was lower in El-Minya than those observed in the previous studies; 98.4% at El-Fayoum, 51.8% in Menoufia and Gharbiya, and 41.7% in Cairo [8,11,13], respectively. These aforementioned differences in seroprevalence may be due to variations in climatic conditions, serological assays used, sample size, breed of sheep, place, and time of sampling [14,20].

Among the evaluated variables, the locality represented one of the risk factors associated with *Toxoplasma* infection. Higher toxoplasmosis seropositivity (p<0.0001) was observed in Matay (57.4% in 94 sheep) compared to that in Dayr Mawas (8.8% in 57 sheep). The significant difference could be attributed to two variables including the difference in the sample size recruited from each district as well as the access frequency of infected cats to sheep pens and grazing areas. In addition, the seroprevalence of infection was significantly (p<0.0001) higher in adult sheep than juveniles. This confirms the previous investigations showing higher rates of exposure to toxoplasmosis infection with increasing age of sheep [11,20-26]. Conversely, no statistically significant effect was observed in relation to abortion history or pregnancy status of the examined sheep and toxoplasmosis.

Indeed, little attention has been devoted to toxoplasmosis surveillance, prevention, and/or treatment in El-Minya. Therefore, we further explored the seroprevalence of *T. gondii* in pregnant women in this area. The overall prevalence was 22.9% in our study, which was lower than those reported in some localities in Egypt; Sharkia (51.5%), El-Fayoum (45.8%), Menoufia (67.5%), 50.8% (El-Minya), 30.2% in six governorates (Alexandria, Beheira, Gharbia, Menoufia, Qalyoubia, and Fayoum), and 33.79% in Menoufia and Gharbiya [7-12], respectively. This fluctuation in toxoplasmosis prevalence among different studies could have been related to environmental conditions, geographical location, humidity, and age distribution among the study population. The incidence of toxoplasmosis infection was previously reported to be higher in humid and warmer climates [27].

In the present study, an increase in *Toxoplasma* seropositivity was associated with increased age of pregnant women. The age effect was insignificant; however, higher odds of exposure in pregnant women of 20 years old and above were observed compared to those of 17-19-year-old group. This observation was consistent with other studies [11,28,29] which could be attributed to the increased odds of infection as age increases.

The impact of two obstetrical factors, namely, parity and gestational age, on acquiring toxoplasmosis in pregnant women was also assessed. Among the two variables, gestational age of the pregnant women had a significant influence (p=0.02) on the toxoplasmosis seropositivity. Pregnant women in their 2^nd^ and 3^rd^ trimesters had higher odds of exposure (OR=19, 95% CI=1-345 and OR=10, 95% CI=0.6-182) compared to those in their 1^st^ trimester. In agreement to our findings, the previous studies in Saudi Arabia, Sri Lanka, and Zambia also documented higher IgG levels in the 2^nd^ trimester compared to the other two trimesters [30-33]. Detection of specific IgG against toxoplasmosis in all trimesters in the present investigation is alarming, especially in those in their 1^st^ and 3^rd^ trimesters. Vertical transfer of toxoplasmosis in the 1^st^ trimester could result in serious consequences in the fetus, whereas higher incidence rates of the parasite in babies are linked to the 3^rd^ trimester due to the rapid transmission pattern of the parasite in that gestational period [34]. Accordingly, routine screening for toxoplasmosis in pregnant women residing in El-Minya Governorate, Egypt, is highly recommended.

Our analysis also indicated a significant association between *T. gondii* infection in pregnant women and contact with cats ranging from 40% in Matay and 46% in Dayr Mawas. This finding was in concordance with those studies showing contact with cats as a risk factor associating human toxoplasmosis [29,35,36]. Cats, the definitive host of *T. gondii*, are a major source of infection for humans and animals due to shedding of oocysts into the environment [37]. Thus, transmission of infection can occur through drinking contaminated water, consumption of contaminated unwashed vegetables and fruits [2]. Hence, the consumption of undercooked infected ovine meat was considered a major risk factor for human infection [38]. The current data further showed that the prevalence of toxoplasmosis was significantly higher in pregnant women who consumed undercooked mutton (kabab and kofta) than those who did not. These data were consistent with those of the previous investigators who showed that consumption of mutton kabab was a significant risk factor for toxoplasmosis seropositivity in pregnant women [8,11,39]. In a previous study in Egypt, undercooked mutton kabab was reported to raise the incidence of toxoplasmosis with the highest percentage of *Toxoplasma* contamination [40]. However, in our retrospective study, we could not definitively determine that undercooked meat was exclusively causal for increased toxoplasmosis in women. In particular, the term undercooked meat is somewhat subjective and, therefore, may skew our data set. It is also not possible to determine if association with cats, exposure to sporulated oocysts in the environment, consumption of undercooked meat, or other food risks such as raw goat milk were the causal factor for individual infections. Therefore, we recognize the limitations of our data in this context. Nevertheless, insignificant association between locality, parity, and exposure to soil and *T. gondii* seropositivity was noted in our investigation. Gelaye *et al*. [41] reported similar findings with an absence of risk factors associating *T. gondii* infection in pregnant women in Ethiopia. Collectively, we do believe our data indicate a strong association between toxoplasmosis and undercooked meat consumption.

## Conclusion

Our data indicate the exposure of both sheep and pregnant women to *T. gondii*, in El-Minya Governorate and the potential role of sheep in transmission of human toxoplasmosis. Special educational programs about preventive and control measures should be implemented. These would include education on the role of cats, undercooked mutton, and consumption of raw goat milk as risk factors for pregnant women. Furthermore, routine toxoplasmosis screening of the pregnant women residing El-Minya Governorate in their 1^st^ and 3^rd^ trimesters is recommended. In addition, further epidemiological investigations are required to determine the rates of toxoplasmosis in other domestic animals, including cats, and other human subgroups including immunocompromised women throughout the governorate. This additional data may be useful in determining the effect of toxoplasmosis on human abortion rates.

## Authors’ Contributions

AEA, MIH, MFNA, and MSR designed the study, shared in sampling, ELISA work, and data analysis. ASMS contributed to sample collection and ELISA work. JJA contributed to literature collection, data analysis, and critical editing and reviewing of the manuscript. All authors wrote, edited, read, and approved the final manuscript.
